# Autophagy in Metabolic Age-Related Human Diseases

**DOI:** 10.3390/cells7100149

**Published:** 2018-09-24

**Authors:** Manon Moulis, Cecile Vindis

**Affiliations:** INSERM, UMR-1048, Institute of Cardiovascular and Metabolic Diseases and University Paul Sabatier; F-31342 Toulouse, France; manon.moulis@inserm.fr

**Keywords:** autophagy, metabolism, diabetes, obesity, liver diseases, atherosclerosis, pharmacological modulators

## Abstract

Autophagy is a highly conserved homeostatic cellular mechanism that mediates the degradation of damaged organelles, protein aggregates, and invading pathogens through a lysosome-dependent pathway. Over the last few years, specific functions of autophagy have been discovered in many tissues and organs; however, abnormal upregulation or downregulation of autophagy has been depicted as an attribute of a variety of pathologic conditions. In this review, we will describe the current knowledge on the role of autophagy, from its regulation to its physiological influence, in metabolic age-related disorders. Finally, we propose to discuss the therapeutic potential of pharmacological and nutritional modulators of autophagy to treat metabolic diseases.

## 1. Introduction

The term “autophagy” was introduced by Christian de Duve, a Belgian cytologist and biochemist who discovered and described the structure and function of lysosomes over 40 years ago [1]. This process is important for maintaining sources of energy at critical times in response to nutrient stress, embryo development, and metabolic functions.

Autophagy exists in three major separate forms: microautophagy, chaperone-mediated autophagy, and macroautophagy. Of these, the most prevalent and best studied form is macroautophagy, hereafter in this review referred to as autophagy. In the general form of the process, cytoplasmic structures targeted for destruction are sequestered within double-membrane vesicles, called autophagosomes, and delivered to the lysosome by fusion for breakdown. The degradation products are transported back to the cytoplasm, where they can be reused for biosynthesis or energy production. In specific conditions, autophagy also selectively targets damaged or unwanted organelles, such as mitochondria, endoplasmic reticulum, and peroxisomes to lysosomes for removal. 

The molecular regulation and machinery of general autophagy has been extensively described in several other reviews, and will only be briefly presented [2,3].

Autophagy is tightly regulated by more than 30 highly conserved genes called autophagy-related (Atg), which were initially characterized in *Saccharomyces Cerevisiae* [4,5,6,7], followed by the discovery of almost all of their mammalian orthologues [8]. Autophagosome formation is induced by metabolic stressors and orchestrated by two major complexes that control the recruitment of specific proteins into newly forming autophagosomal membranes. One complex engaged in the early steps encompasses the unc-51-like autophagy activating kinase 1 or ULK1 (also called Atg1) which interacts with Atg13, Atg101, and the focal adhesion kinase family-interacting protein of 200 kDa (FIP200). Another indispensable complex requires the class III phosphatidylinositol 3-kinase (PI3K) Vps34, which recruits the autophagy specific proteins Beclin-1 (the mammalian ortholog of yeast Atg6), p150/Vps15, Atg14L, or Ambra1 at the assembly sites of the phagophore. The elongation of membranes for the formation of the autophagosome requires two ubiquitin-like conjugating systems. Firstly, the Atg12-Atg5-Atg16L1 system, in which Atg12 is conjugated to Atg5 by Atg7 (similar to an E1 ubiquitin-activating enzyme) and Atg10 (similar to an E2 ubiquitin-conjugating enzyme). Then the conjugated Atg12-Atg5 complex interacts with Atg16L1 and associates with phagophores, where it is localized at the outer membrane of nascent autophagosomes, but dissociates before the achievement of autophagosome formation. Secondly, ubiquitin-like reactions involve the microtubule-associated protein 1 light chain 3 (MAP1LC3/Atg8/LC3). It should be mentioned that mammalian Atg8s can be divided into two subgroups based on their amino acid sequence homology, where LC3A, B, and C constitute the LC3 subfamily, and GABARAP, GABARAPL1, GATE-16 (also known as GABARAPL2), and GABARAPL3 constitute the GABARAP/GATE-16 subfamily. Both LC3 and GABARAP/GATE-16 subfamilies are essential for the autophagic process, acting differentially at early stages of autophagosome biogenesis. Thus far, LC3B is the only Atg8 mammalian ortholog that has been extensively studied and identified as a factor associated with autophagic membranes. The cytosolic form of LC3, LC3-I is generated by the cleavage of pro-LC3 by Atg4 family proteins. LC3-I is then conjugated to the lipid phosphatidylethanolamine (PE) by Atg7 and Atg3 to form LC3-II [9]. Since LC3-II is specifically associated with autophagosomes, the level of LC3-II is correlated with the number of autophagosomes and is considered an indicator of autophagosome formation [10]. LC3 functions as a receptor for the protein p62/sequestosome 1 (p62/SQSTM1), which is a multi-domain adaptor protein located at the autophagosome membranes, and is itself degraded by autophagy [11,12]. Moreover, p62/SQSTM1 has an ubiquitin-binding domain, which serves to link ubiquitinated proteins to the autophagic machinery allowing their degradation.

The mature autophagosomes traffic along microtubules using the dynein-dynactin complex, where autophagosomes fuse with endosomes or lysosomes mediated by an endosomal sorting complexe involving soluble *N*-ethylmaleimide-sensitive factor attachment protein receptors (SNAREs), GTPase Rab7 proteins, and lysosomal-associated membrane proteins, LAMP-1 and LAMP-2. It was recently demonstrated that the transcription factor EB (TFEB), which has an important function in organelle biogenesis and metabolic processes, exhibits a major role in regulating lysosomal function and autophagy. For instance, TFEB can bind to the promotor regions of several autophagy genes, thereby regulating the biogenesis of autophagosomes and the fusion of autophagosomes with lysosomes [13].

In the final step of the autophagic process, the encapsulated “cargo” is degraded by lysosomal proteases and released back into the cytosol [14]. Mounting evidence supports that autophagy exerts a critical influence on multiple human metabolic pathophysiological processes and becomes insufficient in aging organisms. This review provides a summary of our current knowledge on the role of autophagy in metabolic age-related diseases including diabetes, obesity, non-alcoholic steatohepatitis, and atherosclerosis.

## 2. Autophagy in Metabolic Diseases

### 2.1. Diabetes

#### 2.1.1. Pancreatic β-Cell Function and Autophagy

Glycemia is tightly regulated by the pancreatic islets of Langerhans, where β-cells are specialized cells that secrete the hypoglycemic hormone insulin when glycemia increases, leading to glucose uptake in the liver via glycogenesis [15]. Numerous in-vitro and in-vivo studies have demonstrated that autophagy plays a crucial role in the maintenance of β-cell function and survival [16,17]. For instance, an impaired autophagic process results in reduced β-cell mass and pancreatic insulin content, due to increased apoptosis and decreased proliferation of β-cells [18,19,20]. Mice harboring a β-cell-specific deletion of Atg7 (Atg7^f/f^: RIP-Cre) showed Langerhans islet morphological abnormalities and degeneration, p62/SQSTM1 and polyubiquitinated protein accumulation in β-cells, as well as impaired insulin secretion [21]. Thus, under a standard diet, basal autophagy is essential for β-cell function and normal insulin secretion. 

Since pancreatic β-cells undergo extensive proinsulin biosynthesis, a functional endoplasmic reticulum (ER) is critical to convert pro-insulin to the mature form of insulin, facing a high protein-folding burden. Thereby, autophagy through the degradation of misfolded proteins or dysfunctional regions of the ER promotes the homeostasis of β-cells [22,23,24]. It should be noted that the unfolded protein response (UPR) is induced when the ER responds to an overload of unfolded proteins. The resulting ER stress, which consists of protein translation interruption, misfolded protein degradation, and molecular chaperone synthesis, has been described as a stimulus for autophagy. β-cells are also highly susceptible to oxidative stress, and hyperglycemia-induced oxidative stress leads to impaired insulin secretion and β-cell death [25]. Induction of the antioxidant nuclear factor erythroid 2-related factor 2 (Nrf2) in β-cells prevents cellular damage and apoptosis through the transcription of autophagy genes, while its knockout results in decreased β-cell mass [26]. Interestingly, autophagy can also promote Nrf2 activation through p62/SQSTM1-mediated disruption of its interaction with the Kelch-like ECH-associated protein 1 (KEAP1) [27,28]. KEAP1 is a repressor protein that binds to Nrf2 and promotes its degradation by the ubiquitin-proteasome pathway. In addition, autophagy can also prevent oxidative stress through the selective degradation of dysfunctional mitochondria, termed *mitophagy*. However, long-term exposure to glucose and fatty acids, leading to glucolipotoxicity, results in high levels of reactive oxygen species (ROS) overwhelming the mitophagy machinery, and causes the accumulation of dysfunctional mitochondria [29]. Consequently, impaired mitophagy leads to reduced glucose responsive insulin secretion in β-cells [30,31]. Thus, the management of glucolipotoxicity, oxidative and ER stresses, and altered mitochondria by autophagy is essential for β-cell protection.

Recently, it has been described that short-term nutrient deprivation stimulates β-cell crinophagy, a secretory insulin granule-specific autophagic process, while longer starvation is required to induce classical autophagy in β-cells [32,33]. Free fatty acids, including palmitate and cholesterol, stimulate β-cell autophagy, thereby preventing the activation of apoptosis [34,35]. Interestingly, omega-3 fatty acids and vitamin D trigger autophagy in β-cells, which plays a protective role in a mouse model of streptozotocin (STZ)-induced pancreatic β-cell failure [36,37]. Finally, glucagon like peptide-1 (GLP-1) receptor agonists (exendin-4 and liraglutide), as well as a dipeptidyl peptidase-4 (DPP-4) inhibitor (MK-626), have been shown in vitro and in vivo to stimulate β-cell autophagy and improve β-cell function, protecting them against glucolipotoxicity or tacrolimus-induced β-cell death [38,39,40,41]. Therefore, autophagy exerts a critical role in β-cell function and survival under normal conditions, but also acts as an adaptive mechanism in stress conditions by avoiding detrimental effects of β-cell failure and apoptosis.

#### 2.1.2. Autophagy Involvement in Type 2 Diabetes Mellitus 

Diabetes mellitus is a chronic disease affecting more than 420 million people worldwide, characterized by hyperglycemia resulting from a reduced capacity to process glucose. Type 2 diabetes (T2D), also called non-insulin-dependent diabetes, is the most frequent type diagnosed in adults, and occurs as a result of poor diet, physical inactivity, obesity, and ageing, and could often be avoided. The two main pathogenic axes of TD2 are insulin resistance and β-cell failure; however, dysregulated autophagy has been described as an important contributing factor [42]. Insulin resistance is first accompanied by pancreatic β-cell adaptation, with increased β-cell mass and insulin secretion (hyperinsulinemia) to prevent a rise in blood glucose [43]. Nevertheless, these events are followed by β-cell failure and decline, which prevent β-cell adaptation, leading to insufficient pancreatic insulin secretion and impaired insulin action.

An increased number of autophagosomes has been described in rodent β-cells, from insulin-resistant and diabetic models, and human β-cells isolated from islets of T2D patients showed an accumulation of p62/SQSTM1 and autophagic vacuoles [15]. A blockage of β-cell autophagic flux, caused by lysosomal defects in response to chronic glucolipotoxicity, has been proposed as a causal mechanism [33,44,45]. Autophagy impairment could contribute to diabetes development, since in the Atg7^f/f^:RIP-Cre mouse model, two groups described in β-cells a reduced serum insulin level and an impaired glucose tolerance [18,21]. The effect of a global autophagy insufficiency has been evaluated on systemic metabolism and diabetes development. Atg7^+/−^ heterozygote mice displayed no anomalies in their systemic metabolic profile in a basal metabolic state. Similarly to Atg7^f/f^:RIP-Cre mice, Atg7^+/−^ mice develop severe and persistent diabetes only when they are crossed with leptin-deficient (*ob/ob)* mice or fed a high-fat diet (HFD) [46]. This observation is supported by several reports showing that HFD upregulates β-cell autophagy, and conversely the inhibition of β-cell autophagy aggravates deleterious metabolic effects, highlighting the crucial role of autophagy in β-cell adaptation under metabolic stress [46,47,48,49]. In agreement with these results, the overexpression of Atg5 improved the metabolic profile of aged mice, including insulin sensitivity, thanks to an enhanced autophagic activity fighting the increase of metabolic stress [50].

Islet amyloid polypeptide (IAPP), co-secreted with insulin for glycemic regulation, can form extracellular and intracellular aggregates associated with β-cell death. The accumulation of IAPP occurs only in human diabetes, thus suggesting that the role of autophagy in human diabetes would be better than in murine diabetes. To test this hypothesis, transgenic mice overexpressing the human IAPP (hIAPP) were cross-bred or not with Atg7^f/f^: RIP-Cre mice. Only β-cell autophagy-deficient mice developed diabetes, thus showing the important role of autophagy in the protection against hIAPP-induced diabetes [42,51,52,53]. Consistently, the administration of the autophagy enhancer trehalose to HFD-fed mice reduced hIAPP oligomers and islet amyloid deposits, as well as β-cell apoptosis, and ameliorated their global glucose profile [42]. In conclusion, β-cell autophagy is a crucial protective mechanism against the combined effects of metabolic stress and toxic hIAPP oligomers, and its induction could be considered in the treatment of diabetes.

### 2.2. Obesity

#### 2.2.1. Nutrient Status and Autophagy

Autophagy serves as an internal source of stored nutrients under conditions of nutrient limitation. Conversely, food intake leads to a transient increase of plasma amino acid levels, and some of them, especially branched-chain amino acids, including leucine, inhibit autophagy by activating the mechanistic target of rapamycin complex 1 (mTORC1) through a Rheb GTPase-dependent signaling pathway [54,55]. Interestingly p62/SQSTM1 and the leucyl-tRNA synthetase can sense amino acid sufficiency, leading to mTORC1 activation, whereas the GCN2 kinase can conversely sense amino acid deficiency to inhibit mTORC1 [56,57,58]. During short-term fasting, autophagy induction and reduced protein synthesis prevent the depletion of amino acids and lead to an increase in the intracellular glutamine level, while long-term malnutrition depletes amino acid levels [59].

Similarly to amino acids, glucose inhibits autophagy through the Rheb-dependent activation of mTORC1, but also via the insulin-PI3K-Akt pathway, leading to tuberous sclerosis complex 2 (TSC2) phosphorylation and mTORC1 activation [60]. Glucose deficiency is accompanied by a decreased ATP level and a higher AMP/ATP ratio, resulting in AMP-activated protein kinase (AMPK) activation and TSC2 phosphorylation, ultimately leading to mTORC1 inhibition-dependent induction of autophagy [60]. Sirtuin 1 (Sirt1) has also been implicated in the glucose-mediated regulation of autophagy, by mediating forkhead box O1 (FoxO1) deacetylation and upregulation of the small Rab7 GTPase involved in the late autophagosome-lysosome fusion [61,62].

Regarding fatty acids, it has been described in vitro and in vivo that elevated lipid content inhibits autophagosome–lysosome fusion, and that intracellular fatty acid-derived metabolites, such as ceramides and acyl-CoA, also alter autophagy homeostasis [63]. However, some free fatty acids, such as palmitic acid and oleic acid, promote autophagy in an mTORC1-dependent or -independent manner, via c-Jun N-terminal kinase (JNK) and protein kinase C (PKC) signaling pathways [64,65]. In turn, autophagy regulates lipid homeostasis by the selective degradation of lipid droplets (LDs) and avoids lipotoxicity [66].

Nutritional changes also affect autophagy in the adipose tissue, which is known to regulate adipocyte differentiation and adipokine secretion. Hence, overnutrition has been described to dysregulate autophagy, which in turn disturbs adipose tissue homeostasis [67,68,69].

A recent report described that intermittent fasting delays metabolic pathological processes by improving mitochondrial health, DNA repair, and autophagy [70]. Currently, it is assumed that fasting and physical activity promote autophagy, while nutritional excess suppresses autophagy. In accordance, our hunter-gathering ancestors would have had higher levels of autophagy compared to most individuals nowadays, who consume a cholesterol-rich diet combined with a sedentary lifestyle, and therefore risk developing “autophagy deficiency” [71].

#### 2.2.2. Dysregulated Autophagy in Obesity

Obesity is a global health concern given its growing scale, with more than one-third of overweight (BMI 25–29.9 kg/m^2^) or obese (BMI ≥ 30 kg/m^2^) adults worldwide, and its role in the development of chronic metabolic diseases and cancer is well established [72]. Obesity is primary linked to excessive or unbalanced food intake associated with inadequate energy expenditure, due to our sedentary modern lifestyle of high-caloric food intake; however, is also promoted by genetic and environmental factors affecting neurohormonal regulation. Nutrient excess triggers metabolic stress, characterized by increased lipid levels and inflammation, and results in obesity-related complications, such as dyslipidemia, insulin resistance, diabetes, hypertension, fatty liver, and heart disease.

During obesity, autophagy inhibition occurs because of the mTORC1 chronic activation induced by hypernutrition [73,74]. Using genetic and dietary models of obesity, a severe downregulation of autophagy was observed, particularly the expression level of Atg7 in the liver [75]. Autophagy deficiency generally unfavorably influences local or global metabolism, thus promoting a vicious circle of metabolism dysregulation. In contrast, the accumulation of autophagosomes in liver and adipose tissues has been reported in obese patients and genetically or diet-induced obese animal models [73,76,77]. Although mTORC1 activation in response to nutrient excess inhibits autophagy, it is not surprising that increased autophagy is observed in obese expanded adipose tissue characterized by a pro-inflammatory environment, suggesting that upregulated autophagy might serve as a compensatory mechanism. For example, excessive activation of autophagy in adipose tissue could limit excessive inflammation and adipogenesis, whereas autophagy deficiency in the hypothalamus impairs the central control of energy balance [76,78,79]. These observations highlight that the intricate link between obesity and autophagy needs to be further studied.

Mouse models with whole-body or tissue-specific deletion or mutation of autophagic genes display obesogenic metabolic phenotypes, or are predisposed to HFD or genetic-induced obesity [80]. For instance, the specific deletion of Atg7 in murine hypothalamic neurons leads to hyperphagia and obesity, indicating that autophagy plays a crucial role in leptin hunger hormone sensitivity [81]. Interestingly, haploinsufficiency in the key autophagy regulator Atg7 did not directly result in metabolic abnormalities in the absence of nutrient stress, but rather in a predisposition to diabetes in a genetic or diet-induced obesogenic context. This observation underlines that autophagy insufficiency perturbs the metabolic adaptation during stress, thereby facilitating the transition from obesity to diabetes mellitus [46].

Additionally, the epigenetic modulation through coactivator-associated arginine methyltransferase (CARM1)-dependent histone arginine methylation and SUV39H-dependent histone lysine methylation has been shown to regulate autophagy in response to nutrient starvation and HFD-induced obesity, respectively [82,83]. Today, the treatment of obesity and its related health problems is mainly focused on lifestyle modification and behavioral therapy, since the pharmacological management remains challenging [59].

### 2.3. Liver Diseases

#### 2.3.1. Hepatic Metabolism and Autophagy

Autophagy was first evidenced in rodent liver tissues in the 1960s, and early studies in hepatocytes and liver tissue have shown a regulation of autophagy via hormones and amino acids [84]. Macroautophagy, microautophagy, and chaperone-mediated autophagy (CMA) co-exist in the liver. Moreover, the hepatic energy balance is connected to autophagy, since starvation-induced autophagy predominantly takes place in the liver. Using perfused rat livers and in vivo experiments in mice, it has been demonstrated that the autophagic degradation of proteins proceeds at a rate of ∼1.5% of the total liver protein/hour under nutrient-rich conditions, and was enhanced approximately two- to three-fold during starvation. Because the liver is the major organ that produces and supplies blood glucose, the conversion of glucogenic amino acids needed for glucose production has been suggested to be an important metabolic contribution to liver autophagy [85,86]. Indeed, perfusion of a rat liver with glucagon significantly stimulates autophagic protein degradation, and enhances intracellular utilization of glucogenic amino acids through its effect on gluconeogenesis [87]. The liver is the major site for lipogenesis and lipid metabolism, and recently, an alternative pathway of lipid droplet (LD) degradation by autophagy has been revealed in mouse hepatocytes, termed lipophagy [88]. The selective uptake of LDs by autophagosomes contributes to the generation of free fatty acids which are catabolized by β-oxidation and then produce energy and ketone bodies. The precise steps required to initiate and carry out autophagy of the LDs and the molecular adapters involved remain to be elucidated. The small GTPase Ras-related protein Rab7, involved in endocytosis, has been shown to induce the uptake of LDs in nutrient-deprived hepatocytes [89]. Recently, the transcription factor upstream stimulating factor 1 (USF1) has been shown to contribute to abnormal lipid accumulation in the liver by suppressing autophagy via the regulation of mTOR transcription [90].

Most studies have focused on a model in which LDs are sequestered by autophagy and delivered to the lysosome for acid lipase-mediated lipolysis. Recently, the existence of a cooperative mechanism between lipophagy and CMA was suggested, in which the LD-associated proteins, called perilipins, are degraded by CMA. This facilitates lipolysis by cytosolic lipases and assembly of autolipophagosomes for subsequent lysosomal lipid degradation [91]. In light of the critical role of autophagy in liver metabolism regulation, it is not surprising that age-related dysfunctional autophagy has been associated with metabolic diseases, as well as liver-specific disorders. The interplay between autophagy and fatty liver disease will be described in the next section.

#### 2.3.2. Hepatic Autophagy and Fatty Liver/Non-Alcoholic Steatohepatitis

Obesity and T2D are often associated with non-alcoholic fatty liver disease (NAFLD), one of the most common chronic liver diseases in the Western countries. NAFLD encompasses from simple steatosis (non-alcoholic steatosis (NAS)) to steatohepatitis (non-alcoholic steatohepatitis (NASH)) with progressive fibrosis leading to cirrhosis and liver dysfunction. NAFLD is currently considered as the hepatic manifestation of the metabolic syndrome. Because lipophagy participates in selective degradation of LDs, it has been suggested that alterations in autophagy may play a role in the pathophysiology of disorders resulting from excessive lipid accumulation, such as NAFLD. Several genetic interventions have provided evidence that autophagy is a crucial mechanism in liver and metabolic disorders. Mice with a hepatocyte-specific deletion of Atg7 develop hepatomegaly and markedly increased liver triglycerides and cholesterol content, mimicking NAFLD [88,92]. Insulin resistance is thought to be critical to the development of NAFLD, and insulin downregulates autophagy in response to nutrient supplies, but autophagy modulates insulin sensitivity as well. The complex interrelationship between autophagy and both insulin resistance and lipid accumulation has been evidenced by several in vitro and in vivo studies. For instance, in the livers of mice with insulin resistance and hyperinsulinemia, the activity of autophagy and the expression of some key autophagy genes were suppressed. The insulin suppression of autophagy involves the regulatory effect of FoxO1 on the expression of several autophagy genes [93]. Conversely, autophagy activity and levels of Vps34, Atg12, and Gabarapl1 transcripts were increased in the livers of mice with streptozotocin-induced insulin deficiency [93]. Moreover, a severe downregulation of Atg7 expression in the liver was observed in both genetic and dietary models of obesity. While the knockdown of Atg7 resulted in impaired insulin signaling and elevated ER stress, the restoration of the Atg7 expression in the liver dampened ER stress, and enhanced the hepatic action of insulin and systemic glucose tolerance in genetically *ob/ob* mice [75]. Accumulating evidence obtained from human studies indicates that hepatic autophagy flux is impaired in livers from patients with biopsy-proven NAS or NASH, compared to livers from subjects with a histologically normal liver. Furthermore, in NASH patients there is a significant increase in hepatic messenger RNA levels of markers of ER stress (activating transcription factor 4 (ATF4), glucose-regulated protein 78 (GRP78), and C/EBP homologous protein (CHOP)) and autophagy (Beclin-1) compared with NAS patients. Similarly, the protein levels of GRP78, CHOP, and p62/SQSTM1 are significantly elevated in NASH compared with NAS patients [94]. Rubicon, a Beclin-1-interacting negative regulator of autophagosome-lysosome fusion, is shown to be up-regulated in association with autophagy impairment in the livers of mice fed an HFD, as well as in patients with NAFLD. In addition, hepatocyte-specific Rubicon knockout mice displayed significant improvement of liver steatosis on an HFD, as well as attenuation of autophagy impairment in the liver [95]. These findings were substantiated by those of autophagy-promoting pharmaceutical agents like rapamycin, caffeine, or carbamazepine that alleviate liver steatosis [96]. CMA, a critical regulator of lipid metabolism, is also involved in the pathogenesis of liver disease, since hepatocyte-CMA deficient mice spontaneously develop massive steatosis with reduced mitochondrial fatty oxidation and augmented *de novo* lipogenesis [97]. Inflammasome activation is also a common characteristic of NASH, and human livers with steatosis or NASH present decreased TFEB and increased p62/SQSTM1, LC3-II, and NLR family pyrin domain containing 3 (NLRP3) expression. Ezetimibe, a widely prescribed drug for hypercholesterolemia, enhances autophagy flux and concomitantly ameliorates lipid accumulation and apoptosis in palmitate-exposed hepatocytes. In macrophages, ezetimibe blocks the NLRP3 inflammasome pathway in an autophagy-dependent manner and modulates hepatocyte-macrophage interaction via extracellular vesicles. In vivo studies revealed that ezetimibe attenuates lipid accumulation, inflammation, and fibrosis in liver-specific Atg7 wild-type and haploinsufficient mice by autophagy induction through AMPK activation and TFEB nuclear translocation [98]. Recently, human polymorphisms in the immunity-related GTPase family M (IRGM) gene have been associated with an increased risk for NAFLD by affecting autophagy. This strongly supports the role of impaired autophagy in human liver disorders [99]. Summarizing the above studies, hepatic autophagy has major implications in liver diseases associated with metabolic complications.

### 2.4. Atherosclerosis

#### 2.4.1. Vascular Function and Autophagy

The link between vascular function and autophagy has been described in several in vitro and in vivo studies. Vascular endothelial cells (EC) guarantee the normal hemostasis function by secreting the von Willebrand factor (vWF) required for the adhesion of platelets to the injured vessel wall. The secretion of vWF is altered in mice harboring an endothelial cell-specific deletion of Atg7 or Atg5, and a similar alteration in hemostasis is also observed with pharmacological inhibition of autophagic flux [100]. While these results propose that autophagy may regulate thrombosis by controlling vWF secretion, the involved mechanisms remain to be elucidated.

Shear stress is the mechanical force caused by the sliding of the blood on the surface of the endothelium. Multiple EC functions are regulated by shear stress, including gene expression, proliferation, migration, morphogenesis, permeability, thrombogenicity, and inflammation. The induction of autophagy under steady laminar shear stress protects ECs from oxidative stress. In addition, the activation of autophagy up-regulates the expression of the endothelial nitric oxide (NO) synthase and inhibits the expression of the pro-inflammatory and vasoconstrictor peptide endothelin-1 [101,102,103]. Recently, it has been shown that defective endothelial autophagy not only curbs endothelial alignment with the direction of blood flow, but also promotes an inflammatory, apoptotic, and senescent phenotype [104]. The angiogenic function of EC is also regulated by autophagy, since Atg5 silencing reduces tube formation and migration, whereas in contrast the induction of autophagy by over-expression of Atg5 increases angiogenesis. The generation of ROS and the activation of Akt have been suggested as important contributors in the regulation of autophagy-induced angiogenesis [105]. Altogether, these findings demonstrate that autophagy is involved in regulating important endothelial functions, such as hemostasis, vascular tone, and angiogenesis.

Vascular smooth muscle cells (VSMC) possess remarkable plasticity, with the ability to reprogram their expression pattern as contractile, synthetic, osteochondrogenic, and macrophage-like phenotypes. Autophagy has been described as a critical determinant during the process of VSMC plasticity and phenotypic changes in response to cellular stress. Treatment of VSMC with platelet-derived growth factor (PDGF) induces autophagy through an AMPK-independent and mTOR-independent mechanism, resulting in the removal of contractile proteins and the up-regulation of the synthetic phenotype markers osteopontin and vimentin [106]. Notably, autophagy has been recently described to regulate VSMC contraction and relaxation [107]. Vascular calcification is driven by the osteogenic differentiation of VSMC within the vessel wall [108]. Autophagy was identified as a novel adaptive mechanism against phosphate-induced VSMC calcification, by regulating apoptosis and mineralizing matrix vesicle releases from VSMC [109]. Cholesterol loading or oxidized lipid exposure of cultured VSMC triggers the expression of macrophage markers [110] and autophagy induction [109,111]. However, a presumed correlation between the autophagy process and macrophage features of VSMC remains to be elucidated. Within normal unloaded macrophages, cytoplasmic neutral lipases are responsible for LD breakdown. However, in lipid-laden macrophages, it has been proposed that LDs are delivered to the lysosome via an autophagy-dependent process, where they undergo subsequent lysosomal acid lipase-dependent lipolysis [112,113].

#### 2.4.2. Atherosclerosis and Dysregulated Autophagy

Atherosclerosis is a chronic arterial disease and a major cause of vascular death worldwide. The disease is progressive, complex, and often associated with ageing and cardiovascular risk factors, including hypercholesterolemia, hypertension, obesity, diabetes, and cigarette smoking. Atherosclerosis involves the build-up of fibrous and fatty deposits, called plaques, in the vessel wall of large- and medium-sized arteries, ultimately leading to a complex plaque that impedes blood flow. Its major clinical manifestations, such as myocardial infarction, stroke, and peripheral arterial disease, are the result of rupture or ulceration of an “unstable” atherosclerotic plaque [114]. Several autophagy stimuli are present within the developing plaque, such as inflammatory cytokines, ROS, oxidized lipid species, growth factors, and metabolic stress [115]. The classical markers of autophagy, including LC3-II, p62/SQSTM1, and Beclin-1, have been identified by immunoblot and immunofluorescence microscopy analysis in both human atheroma lesions and in ApoE-null mice that spontaneously develop atherosclerosis [116]. Interestingly, the progression of atherosclerotic plaques in ApoE-null mice is accompanied by autophagy deficiency, as assessed by the accumulation of p62/SQSTM1 in atherosclerotic aortas. The general consensus is that successful autophagy of damaged components has a beneficial effect against atherogenic stressors and promotes plaque cell survival [115]. Loss of EC or VSMC participates in the thinning of the fibrous cap, resulting in plaque fragilization and rupture. Interestingly, the exposure of EC to oxidized LDL leads to an autophagy process involving surface expression of lipid phosphatidylserine, an “eat-me” phagocytic signal, via a mechanism involving Beclin-1 [117,118]. Consequently, it is assumed that autophagy is anti-atherogenic by favoring cell survival, oxidizing LDL processing, and the clearance of pro-thrombotic apoptotic cells. More recently, using gain- or loss-of-expression approaches, it has been reported that mitophagy plays a major role in VSMC fate after exposure to oxidative and metabolic stress by favoring cell survival or enhancing apoptosis, respectively. Indeed, PINK1 or Parkin knockdown by small-interfering RNAs, increases the cytotoxic response of human VSMC challenged with oxidized LDL, whereas PINK1 or Parkin overexpression has cytoprotective effects [119]. The importance of autophagy in atherosclerosis progression was further demonstrated in ApoE-null mice with specific VSMC deletion of Atg7 that exhibited accelerated atherosclerotic plaque development after 10 weeks of HFD, as shown by increased plaque cell death, inflammation, and fibrous cap thinning [120,121]. The absence of autophagy in ApoE-null mice harboring a conditional deletion of Atg5 within macrophages enhances plaque formation and leads to macrophage inflammasome hyperactivation accompanied by increased interleukine-1β production [122]. Similarly, defective macrophage autophagy led to increased apoptosis and oxidative stress in advanced lesional macrophages, promoted plaque necrosis, and worsened phagocytic clearance in Atg5-deficient macrophages/Low density lipoprotein receptor (LDLR)-null mice [123]. Recent data have shown that Atg5-null macrophages developed further p62/SQSTM1 accumulation at the sites of large cytoplasmic ubiquitin-positive inclusion bodies. Aortas from atherosclerotic mice and plaques from human endarterectomy samples displayed an increased abundance of p62/SQSTM1 and polyubiquitinated proteins that colocalized with plaque macrophages [124]. Together, these data strongly support that the dysregulation of autophagy may be a contributing factor in the progression of atherosclerosis.

## 3. Pharmacological Modulation of Autophagy in Metabolic Age-Related Diseases

Currently, no pharmacological intervention aimed at specifically modulating autophagy is available for use in human disorders. However, some metabolic and hepatic disorders have been demonstrated to respond to behavioral and pharmacological autophagy-modulatory interventions [125]. The activation of autophagy by caloric restriction or physical training improves insulin sensitivity in old rats [126] and in obese mice [127]. Caloric restriction mimetics (CRMs), which would pharmacologically mimic the beneficial effects of caloric restriction or fasting, have gained increasing attention. For example, metformin, the first-line antidiabetic drug, activates AMPK and induces autophagy. Interestingly, both caloric restriction and metformin significantly improve body weight and glucose homeostasis, along with hepatic steatosis in ob/ob mice by stimulating autophagic flux [128]. Similarly, the CRM resveratrol potentiates the effect of a low dose of rapamycin, and the combination decreases obesity and prevents hyperinsulinemia in male mice on HFD [129]. Berberine (BBR), an isoquinoline plant alkaloid, displayed glucose- and cholesterol-lowering properties. Interestingly, BBR has been shown to reduce glycemia and plasma cholesterol in diabetic patients, and has been hypothesized that its anti-hyperglycemic activity involves enhanced AMPK signaling, thus promoting autophagy [130,131]. The natural polyamine spermidine has prominent cardioprotective and neuroprotective effects in aged rodent models [132]. Daily administration of the polyamine spermine to mice fed an HFD prevented adiposity and improved glucose tolerance [133]. Thus, supplementation with CRMs could be a new therapeutic strategy for diabetes or a metabolic syndrome with lipid overload. Recently, a novel autophagy enhancer MSL (4-(4-fluorophenyl) sulfonyl-5-methylthio-2-phenyloxazole), was identified by high-throughput screening, and it displays increased LC3-I to LC3-II conversion without mTOR inhibition. MSL accelerates intracellular lipid clearance, and its administration improved the metabolic profile of ob/ob mice and ameliorated inflammasome activation [134]. Furthermore, the disaccharide trehalose invokes the activation of autophagic flux. Its mechanism of action has been recently uncovered and involves TFEB-dependent thermogenesis in hepatocytes, concomitant with the upregulation of hepatic and white adipose tissue expression of UCP1 (uncoupling protein 1 (mitochondrial, protein carrier)) [135]. Ezetimibe, a drug approved by the Food and Drug Administration (FDA) for the treatment of hypercholesterolemia, improves NAFLD and alleviates oxidative stress by the activation of AMPK, which in turn phosphorylates p62/SQSTM1 [136]. The pharmacological inhibition of calcium channels using the FDA-approved drug verapamil successfully activates autophagic flux and reduces body weight, hepatic LD accumulation, insulin resistance, and steatohepatitis of HFD-fed obese mice, suggesting that calcium channel blockers can be used to treat general NAFLD pathologies [137]. The autophagy inducer rapamycin and its derivatives (everolimus), which act via the inhibition of mTOR, have been tested as plaque-stabilizing compounds. Interestingly, when everolimus is administrated by a local stent in cholesterol-fed rabbits, a remarkable reduction in the content of macrophages, but not in the amount of VSMC, was observed [138]. The inhibition of translation by dephosphorylating the downstream mTOR target p70 S6 kinase is associated with a selective apoptotic cell death of macrophages, a huge degradation of long-lived proteins, the processing of LC3, and a cytoplasmic vacuolization. Therefore, since macrophage efferocytosis and autophagic flux decline, along with the progression of atherosclerosis [122,123], the selective clearance of lesional macrophages via everolimus-induced autophagy might be a promising therapeutic strategy to stabilize atherosclerotic plaques. Nevertheless, therapy using mTOR inhibitors is correlated with adverse side effects, including hypercholesterolaemia and hyperglycemia, which can negatively affect plaque stability [113]. In addition, chronic rapamycin treatment, at a dose used to extend lifespan, impaired hepatic glucose homeostasis by the induction of gluconeogenesis, an effect mediated by mTORC2 disruption [139]. Taken together, these observations confirm the valuable therapeutic interest of the use of pharmacological and nutritional autophagy activators in patients with metabolic age-related disorders.

## 4. Conclusions

**Figure 1:**
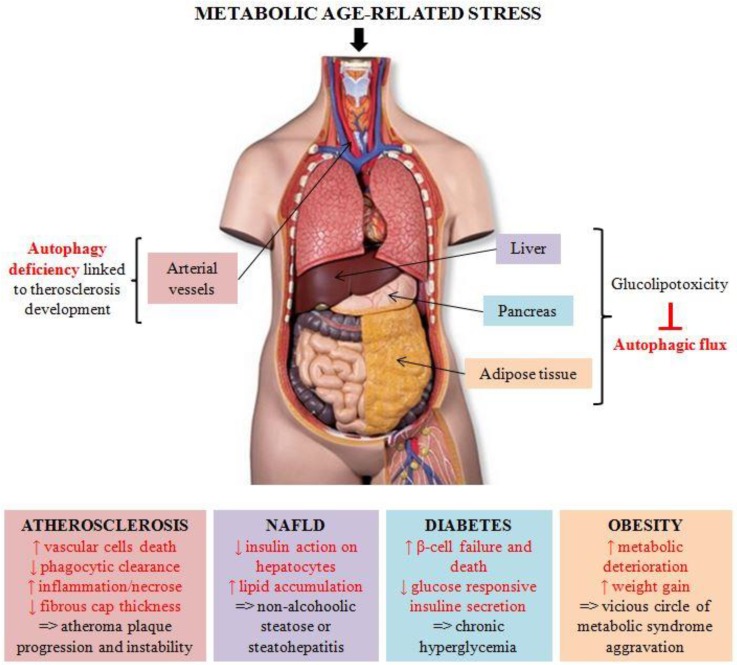
Autophagy in metabolic-age related diseases. Metabolic age-related stress triggers metabolic syndrome, characterized by obesity or metabolic complications, including atherosclerosis, non-alcoholic fatty liver disease (NAFLD), and diabetes mellitus. In all these pathologies, alterations in the autophagic process seem to play a crucial role in disease onset and progression.

In this review, we have described the crucial involvement of autophagy in metabolic homeostasis and its important role in many metabolic-age related disorders (Figure 1). Despite the availability of numerous cell-based and animal models that provided fundamental insights into the autophagic process, additional studies are required to fully understand the tissue-specific regulation of autophagy during ageing. Since autophagy declines with age, the development of therapeutic strategies tackling this age-related deficiency is a promising field of research.
